# In the tracks of a whale: inferring size class, orientation and swimming speed from thermal flukeprints

**DOI:** 10.1242/jeb.251816

**Published:** 2026-04-17

**Authors:** Lucie Laporte-Devylder, Henrik Skov Midtiby, Magnus Wahlberg

**Affiliations:** ^1^ Department of Biology, University of Southern Denmark, Campusvej 55, DK-5230 Odense, Denmark; ^2^ UAS Center, Maersk McKinney Møller Institute, University of Southern Denmark, Campusvej 55, DK-5230 Odense, Denmark

**Keywords:** Thermal infrared imaging, Flukeprints, Cetacean conservation, Non-invasive monitoring, Drone

## Abstract

Despite decades of innovation, monitoring whales remains constrained by their brief surfacings, wide-ranging movements and limitations of conventional survey methods. Unmanned aerial vehicles (UAVs) and thermal infrared (TIR) sensors offer non-invasive alternatives, yet most applications rely on short-lived direct detections. Here, we evaluated a novel approach using UAV-based TIR imaging to extract biological and behavioural information from thermal flukeprints, surface disturbances generated by whale tailbeats. Field surveys of free-ranging humpback whales (*Megaptera novaeangliae*) off Reunion Island demonstrated that flukeprint width robustly distinguished calves from adults. Flukeprint spacing provided swimming-speed estimates consistent with RGB-based measurements (mean error 0.42 m s^−1^; median relative error 16%), while flukeprint-derived orientations aligned closely with visual headings (mean offset −3.1 deg). These results indicate thermal flukeprints capture age class, movement and directional information even when animals are partially visible or submerged. By extending observations beyond direct sightings, TIR-detected flukeprints offer a low-impact, complementary tool for cetacean monitoring.

## Teaser

**Summary:** Drone-captured thermal flukeprint patterns left by whales enable estimation of age class, swimming speed and heading even after animals submerge, offering a complementary tool for non-invasive monitoring.

## INTRODUCTION

Monitoring marine mammals is essential to understand population dynamics, migration patterns, habitat use and responses to anthropogenic stressors, knowledge that underpins both effective conservation and sustainable ocean management (Booth et al., 2020). Despite recent progress in available methods and technologies, monitoring marine mammals in the wild remains challenging. Their highly mobile nature and sporadic surfacing behaviour, and the vast, dynamic marine environment they inhabit make observation efforts difficult. Traditional monitoring methods, such as boat-based surveys, aerial overflights, direct visual tracking and animal-borne data loggers, are logistically complex, financially costly, weather dependent and, in some cases, risk altering the animals' natural behaviour (Pabst et al., 2000; Smith et al., 2020; Verfuss et al., 2018).

In recent years, the rapid development of unmanned aerial vehicle (UAV) technologies, in parallel with advances in imaging sensors, have opened new avenues for more efficient, scalable and less intrusive wildlife monitoring in both terrestrial and marine ecosystems (Kline et al., 2025; Koger et al., 2023; Linchant et al., 2015; Yaney-Keller et al., 2025). Drones equipped with high-resolution RGB or thermal infrared (TIR) cameras have been increasingly employed for direct detection of animals, offering improved vantage points and reduced disturbance (Brunton et al., 2020; Gazagne et al., 2024; Larsen et al., 2023; Perryman et al., 1999; Pinel-Ramos et al., 2025; Raoult et al., 2018; Richter et al., 2023 preprint; Yaney-Keller et al., 2025). However, thermal detection remains constrained by short observation windows, typically limited to brief surfacing events, as well as environmental factors such as glare, cloud cover and sea state that can obscure visibility or reduce detection reliability (Baldacci et al., 2005; Guazzo et al., 2019; Smith et al., 2020; Zitterbart et al., 2020).

**Fig. 1.**
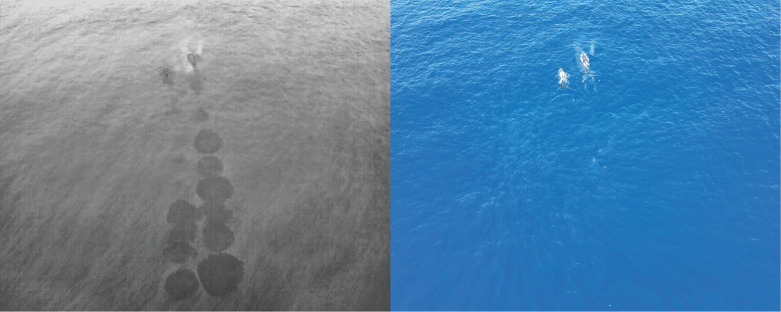
**Synchronized drone imagery of two humpback whales (*Megaptera novaeangliae*) showing complementary information from thermal infrared (TIR) and RGB sensors.** Left: TIR image revealing flukeprints trailing each individual. Right: RGB image showing the whales' body outlines.

To address these constraints, one promising strategy is to shift focus from direct sightings to indirect signs of presence. In terrestrial ecology, the analysis of animal tracks left in soft substrates such as snow, sand or mud is a well-established method for inferring movement patterns, species identity, group size and behaviour (Dorfman et al., 2023; Finerty et al., 2024). In the marine environment, a similar kind of indirect evidence of whale activity comes in the form of flukeprints, which are surface water disturbances generated by the propulsive motion of a cetacean's fluke during subsurface swimming (Fig. 1). From a hydrodynamic standpoint, flukeprints result from a combination of vortex shedding, turbulence and vertical displacement of water masses, which disrupt the local surface roughness and tension (Han et al., 2020; Levy and Uminsky, 2012; Levy et al., 2011; Rousseaux, 2012 preprint). These disturbances typically form smooth, elliptical patches that contrast with the surrounding wind-roughened sea surface. Although often subtle or transient in RGB imagery (Cubaynes et al., 2019), flukeprints are substantially more visible in the thermal infrared spectrum (Churnside et al., 2009; Florko et al., 2021; Lonati et al., 2025). This enhanced detectability arises from temperature differentials between the displaced subsurface water and the ambient surface layer (Florko et al., 2021; Levy et al., 2011). While flukeprints traditionally refer to disturbances caused by tail strokes, thermal imagery can additionally capture broader body-associated thermal marks left when the whale surfaces (Fig. 1).

TIR imagery has revealed the systematic presence of cetacean flukeprints in aerial surveys, suggesting their utility as passive, indirect cues for detecting whale presence, even when the animals themselves are not visible (Florko et al., 2021; Horton et al., 2019). Thermal flukeprints persist longer than visual cues, potentially offering complementary possibilities for observation (Churnside et al., 2009; Florko et al., 2021). Despite their potential, flukeprints remain an underexplored phenomenon, and systematic research is lacking to determine whether their characteristics can be reliably linked to behavioural or biological metrics.

This study evaluated the potential of thermal flukeprints as a tool for cetacean monitoring. We focused on free-ranging humpback whales (*Megaptera novaeangliae*) at a breeding site off the western coast of Reunion Island in the Indian Ocean. We tested the hypothesis that thermal flukeprints can be used to extract meaningful biological and behavioural metrics from wild whales. Specifically, we assessed whether thermal flukeprints can: (1) support size class identification by distinguishing between adult and juvenile individuals, (2) serve as indicators of swimming speed estimates and (3) provide information on the orientation and short-term movement direction of individual whales, based on consecutive flukeprints.

These parameters are typically difficult to obtain from RGB imagery alone, particularly when individuals surface briefly or only partially. By validating the informational content and persistence of thermal flukeprints, this research aimed to establish a low-impact, complementary method for drone-based cetacean monitoring.

## MATERIALS AND METHODS

### Study sites and survey periods

Humpback whales, *Megaptera novaeangliae* (Borowski 1781), aggregate seasonally in tropical and subtropical coastal waters during the austral winter to engage in breeding and calving activities. These low-latitude breeding grounds are typically characterized by shallow, warm waters where whales exhibit prolonged surface behaviour associated with courtship, maternal care and social interaction (Kavanagh et al., 2017). Such conditions provide a favourable context for evaluating indirect detection methods, including thermal flukeprint analysis, as frequent and sustained surface activity increases the likelihood of detecting surface-generated traces and facilitates temporal matching between visual observations and thermal signatures.

Field surveys were conducted off the western coast of Reunion Island (Fig. S1) in the southwestern Indian Ocean (21°S, 55°E) between 18 July and 15 August 2024. This period aligns with peak seasonal presence of humpback whales belonging to Breeding Stock C (International Whaling Commission, 1998). The population during this time primarily consists of sexually active adults, mature males and females with calves, many of which exhibit prolonged surface-associated behaviour.

The study area spans a narrow coastal shelf with warm sea surface temperatures and generally calm conditions, enabling consistent and stable aerial monitoring. Local vessel traffic varies across zones of both low and moderate anthropogenic activity. These environmental and behavioural characteristics supported the controlled assessment of thermal flukeprint visibility, structure and persistence, while allowing for direct visual confirmation of individual animals.

### Survey protocol and image acquisition

Aerial data were acquired using a DJI Mavic 3 Thermal (M3T), a compact quadcopter equipped with integrated dual-sensor payloads for simultaneous visual and thermal monitoring. The system includes a high-resolution RGB camera (48 MP, ½ inch CMOS sensor) and a TIR sensor with a resolution of 640×512 pixels, operating in the 8–14 µm longwave infrared band at 30 Hz with a thermal sensitivity of ≤50 mK (at f/1.0). Both sensors were gimbal stabilized, enabling a downward-facing camera orientation perpendicular to the sea surface (i.e. a 90 deg nadir view), which minimizes motion blur and standardizes geometric projection. This configuration allowed concurrent acquisition of RGB and TIR data for complementary detection of surface cues such as flukeprints and direct visual confirmation of whale presence and morphology.

**Fig. 2.**
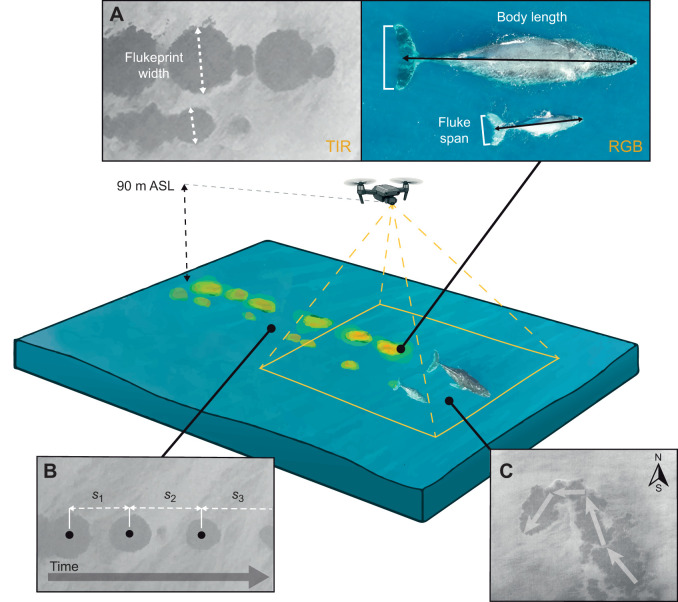
**Schematic representation of a mother–calf pair of humpback whales swimming, illustrating the parameters quantified from multi-sensor unmanned aerial vehicle (UAV) imagery.** (A) Body size and age class derived from flukeprint width. (B) Swimming speed calculated from flukeprint spacing where *S_x_* is the distance between two successive flukeprint centroids. (C) Orientation determined from flukeprint alignment. The UAV is shown at 90 m ASL, with its field of view covering the whales and their associated thermal flukeprints visible at the surface of the water.

Flights were conducted at a target altitude of 90 m above sea level (ASL), selected to balance spatial resolution and field of view while remaining within the European Union Aviation Safety Agency (EASA) Open Category ceiling of 120 m. Prior to take-off, the UAV maximum altitude was manually set to 90 m and maintained throughout focal follows. Altitude was monitored using onboard barometric telemetry with continuous live feedback to the remote controller, and under typical survey conditions, altitude variance was within ±0.5 m. All imagery was initially captured at the nadir to ensure consistent geometry and accurate detection and measurement of surface traces (Fig. 2), with oblique viewing angles introduced in later flights to assess whether increased angular coverage improved temporal or spatial resolution.

Whales were visually located prior to launch using binoculars or surface observations from shore- or vessel-based platforms and drones were deployed only when animals were within 500–900 m of the launch point. Flights were limited to 5–20 min to minimize aerial presence and potential habituation. All operations followed a standardized, non-invasive survey protocol designed to minimize disturbance while maximizing thermal signature detectability. Drones approached animals laterally or from the rear, avoiding frontal or overhead trajectories, and local regulatory minimum approach distances were strictly observed (Arrêté Préfectoral, 2021; no. 2021-1306, Annexe 2). Flights were conducted well above the commonly recommended minimum altitude for UAV-based cetacean monitoring (25 m ASL; Aubin et al., 2023; Torres et al., 2018) and manufacturer-provided lower-noise propellers, compliant with EU C2 noise class requirements for small UAVs (≤82 dBA), were used to further reduce potential acoustic disturbance.

All data were collected using non-invasive observational methods under permits issued by the Direction de l'Environnement, de l'Aménagement et du Logement (DEAL) Réunion and the Réserve Naturelle Marine de La Réunion (decision no. DEAL/SEB/UBIO/2024-69). Visual observers monitored whale behaviour throughout all flights, with predefined criteria for immediate flight termination in the event of potential disturbance (e.g. abrupt changes in swimming direction, sudden dives, prolonged submergence or increased movement speed). No significant behavioural responses attributable to drone presence were observed and animals generally continued their activity undisturbed. The UAV observation protocol was reviewed and approved by the Research Ethics Committee of the University of Southern Denmark (approval no. 25/66344).

During each flight, time-synchronized RGB and TIR videos of swimming whales and their associated flukeprints were collected (Fig. 2). RGB imagery served as the primary observational reference (‘ground truth’) against which thermal-based detections and measurements were calibrated and validated. Whale positions, surfacing events and fluke dimensions were first identified in RGB frames before assessing corresponding measurements in TIR imagery, providing a robust multi-modal dataset for evaluating flukeprints in relation to whale behaviour, individual traits and environmental conditions.

### Camera calibration and measurement accuracy

To extract quantitative biological and behavioural parameters from drone imagery, we used the software Drone Video Measure (DVM), an open-source photogrammetric and motion analysis software (github.com/egemose/DroneVideoMeasure). The program allows frame-by-frame annotation, measurement extraction and spatial referencing of drone footage using geotagged metadata.

Prior to data extraction, both the RGB and TIR sensors were systematically calibrated within DVM. Geometric calibration of the RGB camera was performed using a checkered board of known dimensions filmed from multiple angles, allowing the software to estimate intrinsic camera parameters and correct for lens distortion. Thermal camera calibration followed a similar procedure, using identifiable fixed targets of known size visible in the thermal spectrum. These calibrations ensured accurate pixel-to-distance scaling and spatial alignment between image coordinates and real-world measurements for both sensor modalities.

To validate measurement accuracy at the operational survey altitude (90 m ASL), we conducted ground-truth tests (Table S1, Fig. S2) using a research vessel of known length (5.5 m). The vessel was filmed under the same flight conditions as whale surveys and lengths measured in DVM were compared with the true physical length to quantify absolute measurement error. Drone altitude was recorded using the onboard barometric altimeter and scaling against the reference vessel accounted for any flight-to-flight variation in altitude. Across repeated measurements, DVM slightly underestimated object length, with a mean absolute error of 0.15 m (≈2.8%) and 0.16 m (≈2.8%) for RGB and TIR imagery, respectively. In addition to length, the vessel's motion was used to evaluate orientation and speed estimates derived from drone imagery. Portions of video showing the vessel moving at a constant speed of 4.1 m s^−1^ (≈8 knots, verified via onboard GPS logging) were analysed. The bow of the vessel was tracked frame by frame in both RGB and TIR imagery to measure orientation and velocity. DVM-derived estimates closely matched the known speed and heading, with speed errors within ±0.4 m s^−1^. Swimming speed was calculated from frame-to-frame displacement of the object of interest in the calibrated image plane and scaled to real-world distance using the photogrammetric calibration and flight altitude. Drone GPS coordinates were used only for georeferencing of footage and not for displacement calculations. Previous validation studies have shown that velocities derived from drone-based tracking in DVM closely match GPS-measured speeds of moving vessels (Brennecke et al., 2022). These validation steps confirm that the measurement workflow provides accurate length, orientation and motion estimates under operational survey conditions and the same calibration procedure was applied to whale imagery to minimize systematic measurement bias.

### Morphometrics measurements

Once calibration and validation procedures were complete (see ‘Camera calibration and measurement accuracy’, above), biological measurements were extracted from selected video segments (Fig. 2). Size measurements of humpback whales were obtained during surface intervals when the full body was visible and aligned with the water surface. Linear body length was estimated as the distance from the tip of the rostrum to the fluke notch, following established photogrammetric approaches (Christiansen et al., 2016). Fluke span was measured as the distance between the distal tips of the flukes when clearly extended at the surface, typically immediately prior to a dive. Image scaling was derived from the calibrated camera models and field validation procedures described in ‘Camera calibration and measurement accuracy’ (above), rather than from independent altimeter-based corrections. This approach has been shown to provide sufficient accuracy for cetacean morphometric measurements under comparable UAV survey conditions (Ratsimbazafindranahaka et al., 2021).

Thermal flukeprints associated with identified surfacing events were measured for each whale within a standardized temporal window following fluke passage. Only prints captured at the earliest clearly defined boundary (≤5 s after fluke emergence) were included in size analyses. This window represents a compromise between maximizing wake visibility and minimizing deformation due to diffusion and expansion. Prints that were visibly degraded or distorted were excluded from size estimation but retained for movement analysis. Flukeprint width was defined as the axis parallel to a whale's fluke and perpendicular to its swimming direction (Fig. 2).

### Body size and age class

To evaluate the information content of thermal flukeprint dimensions, we assessed whether flukeprint width reflects broad age-class differences in body size and continuous variation in body size within age classes. Mean flukeprint width, body length and fluke span were quantified for individually identified whales and summarized per individual to account for repeated measures. Individuals were assigned *a priori* to age classes (calf or adult) based on independent visual criteria derived from RGB imagery (Fig. S6). Calves were defined as individuals consistently observed in close spatial association with an adult female and estimated to be ≤50% of the adult's body length; all other individuals were classified as adults. Age-class assignments were made prior to thermal analysis and were not informed by flukeprint metrics. We first fitted linear regression models relating mean flukeprint width to mean body length and mean fluke span across all individuals to provide a descriptive overview of scaling patterns. To test whether scaling relationships differed between calves and adults, we fitted models including age class and its interaction with flukeprint width. In addition, we performed separate regressions within each age class to evaluate whether flukeprint width contains information about body size beyond categorical age differences. Model performance was assessed using 10-fold cross-validation, calculating root mean squared error (RMSE) and coefficient of determination (*R*^2^) on held-out data. To assess the ability of flukeprint width to discriminate age classes, we fitted a logistic regression model with age class as the response and mean flukeprint width as the predictor. Classification performance was evaluated using receiver operating characteristic (ROC) analysis, and the optimal threshold was determined using Youden's *J* statistic. Predicted probabilities were converted to class assignments to generate confusion matrices and quantify classification accuracy.

### Swimming speed

To evaluate consistency between swimming speed estimates derived from RGB and TIR imagery, we calculated mean speed for each whale and surfacing event using consecutive frame-to-frame displacements. RGB and TIR videos were acquired simultaneously at identical frame rates (30 Hz), ensuring matched temporal resolution across sensors. Analyses were restricted to surfacing sequences of continuous, linear swimming, while behaviours involving abrupt changes in velocity or orientation (e.g. breaches, pectoral slaps, escape manoeuvres) were excluded. From RGB imagery, whale position in each frame was defined by the manually identified rostrum tip, which remained consistently visible throughout the swimming sequences and provided a stable anatomical landmark for tracking an individual across frames. Frame-to-frame Euclidean displacement was divided by elapsed time to estimate instantaneous speed, and values were averaged within each surfacing event. From TIR imagery, swimming speed was inferred from the spacing of thermal flukeprints, defined as discrete thermal signatures left by the whale's fluke at the surface. Only flukeprints of this type were retained to avoid bias from additional body impressions or subsurface propulsion thermal signatures (see examples in Fig. S7). The distance between centroids of successive flukeprints along a linear trajectory was divided by the corresponding time interval, with mean inter-centroid spacing used when three or more consecutive prints were available. For each surfacing event, speed estimates were calculated from multiple consecutive measurements and averaged to obtain a single mean speed per event; mean speeds derived from RGB and TIR imagery for the same surfacing event were then paired for comparison.

Agreement between RGB- and TIR-derived speed estimates was assessed using complementary metrics. Mean bias and 95% limits of agreement were calculated, and overall error was quantified using RMSE, mean absolute error (MAE) and normalized RMSE (nRMSE, scaled by mean observed speed). Relative error was assessed using mean absolute percentage error (MAPE) and median MAPE to provide both average and typical estimates of proportional discrepancy. Reliability was evaluated using the intraclass correlation coefficient (ICC; two-way random effects model, absolute agreement). A paired *t*-test was used to detect any systematic differences between methods. Agreement was visualized using Bland–Altman plots and proportional bias was assessed by regressing paired differences against mean speed. Representative examples of surfacing events are provided in Fig. S7. The relationship between mean flukeprint spacing and swimming speed was examined using TIR-derived data only. A log–log linear mixed-effects model (LMM) was used to predict mean swimming speed from mean flukeprint spacing, with whale identity included as a random intercept to account for repeated measurements. Body length was evaluated as an additional fixed effect but was excluded from the final model when it did not improve model fit (Table S2). The final model can be expressed as: log(speed*_ij_*)=β_0_+β_1_log(spacing*_ij_*)+*u_i_*+ε*_ij_*, where *u_i_*∼*N* (0, σ^2^) represents the random effect for whale *i* and ε*_i_*∼*N* (0, σ^2^) is the residual error associated with surfacing event *j*. Model assumptions were assessed via inspection of residuals. Model explanatory power was quantified using marginal and conditional *R*^2^. Predictive performance of the model was evaluated using leave-one-whale-out cross-validation (LOOCV), whereby all surfacing events from a given individual were withheld during model fitting and subsequently predicted. Predictions were generated in log space and back-transformed to the original scale. Model performance was assessed using RMSE, MAPE marginal and conditional *R*^2^ and the proportion of observed speeds falling within 95% prediction intervals. Calibration and potential systematic bias were assessed using Deming regression by testing whether the slope differed from unity and the intercept from zero.

The validated spacing–speed relationship was then applied to TIR still images that were not included in the model-fitting or cross-validation datasets. For still images in which at least two consecutive flukeprints were visible, flukeprint spacing was measured and swimming speed was predicted using the fixed-effect estimates from the log–log LMM. Predictions were generated in log space and back-transformed, reporting both median and bias-corrected mean estimates. Prediction uncertainty was quantified by propagating fixed-effect uncertainty, random-intercept variance and residual variance to obtain 95% prediction intervals. Predicted speed from still images was compared against mean swimming speed independently derived from RGB video sequences corresponding to the same surfacing events.

### Orientation

To evaluate the reliability of TIR flukeprints as indicators of whale orientation, we compared TIR-derived surface headings with headings obtained from synchronized RGB video imagery, which provided a direct visual reference of body axis (tail-to-rostrum vector). Orientation data were extracted frame by frame, and TIR and RGB observations were paired by whale identity, surfacing event and flukeprint ID (i.e. for each surfacing event, individual flukeprints were assigned a sequential identifier). For each flukeprint, the posterior–anterior axis was determined from surface morphology in the TIR imagery. Flukeprints appeared as oval surface disturbances elongated along the whale's swimming axis. The posterior edge typically exhibited earlier and stronger diffusion, whereas the anterior edge retained a sharper boundary (Fig. S8). A vector was drawn from the posterior to the anterior edge and the compass bearing of this vector was taken as the TIR-derived heading. Corresponding RGB headings were calculated from vectors connecting the tail and rostrum of tracked whales. All headings were expressed on a 0–360 deg geographic compass scale. Agreement between TIR- and RGB-derived headings was evaluated using circular statistics. Headings were converted to circular variables, from which circular mean, standard deviation and correlation coefficient were computed. Paired angular differences were calculated in the −180 to 180 deg range and analysed at both the individual and pooled levels. Differences were classified into agreement categories (Excellent, ≤5 deg; Good, ≤15 deg; Acceptable, ≤20 deg; Discrepant, *>*20 deg). A Rayleigh test was applied to assess clustering of angular differences. Accuracy was further summarized as mean heading error, circular standard deviation and the proportion of estimates with *<*20 deg error.

## RESULTS AND DISCUSSION

Across 116 videos analysed, flukeprints were detected in all surfacing events, providing a consistent basis for measurement of size, speed and orientation. To evaluate the performance of thermal flukeprint analysis as a monitoring tool, results are presented together with their interpretation and methodological implications. For each parameter, we first report quantitative validation against RGB-derived reference measurements and then discuss the biological and technical factors influencing accuracy and applicability.

### Body size and age class

**Fig. 3.**
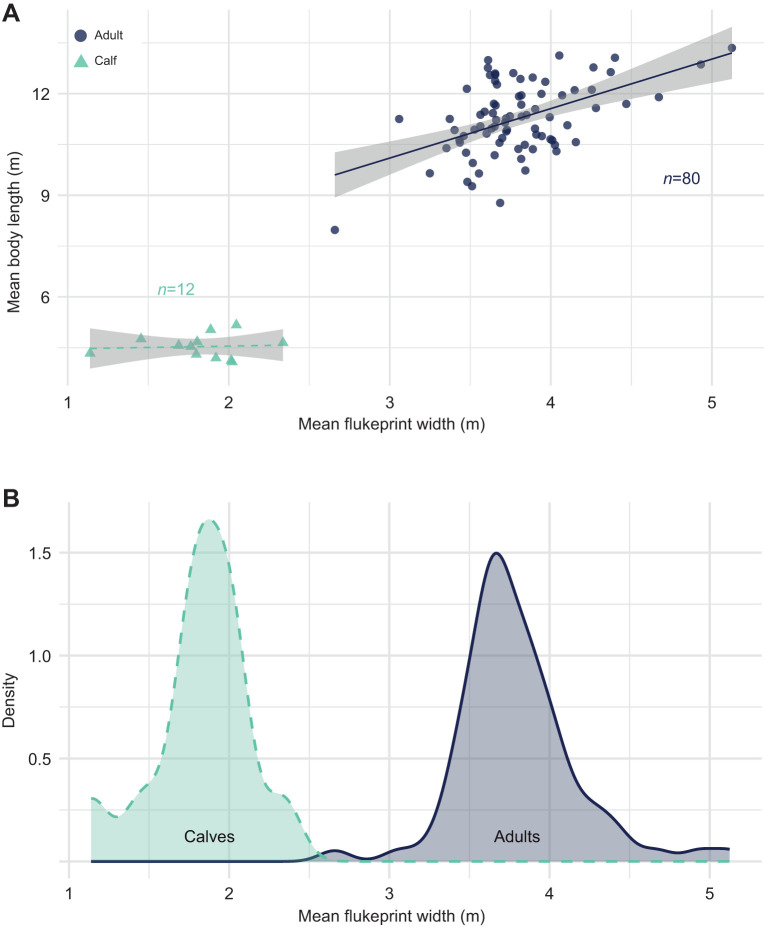
**Size analysis of humpback whales from UAV imagery.** (A) Separation of body length into distinct size classes based on flukeprint width for *n*=92 whales. (B) Mean flukeprint width for calves and adults showing clear separation between the two age classes.

Across all individuals (*n*=92, adults and calves pooled), mean flukeprint width was strongly and positively associated with both mean body length and mean fluke span (Fig. 3A). A simple linear model indicated that flukeprint width explained 81% of the variance in body length (adjusted *R*^2^=0.81, slope=2.95±0.15, *t*_90_=19.49, *P*<0.001) and 71% of the variance in fluke span (adjusted *R*^2^=0.71, slope=0.88±0.06, *t*_90_=14.98, *P*<0.001). Intercepts did not differ significantly from zero for either relationship (body length: *P*=0.94; fluke span: *P*=0.65). Visual inspection of the data, however, revealed two distinct clusters corresponding to calves and adults, indicating that the strong global relationships primarily reflect categorical differences between age classes rather than continuous scaling across all individuals (Fig. 3A).

To formally test whether scaling relationships differed between age classes, we fitted linear models including age class and its interaction with flukeprint width. For both body length (Fig. 3A) and fluke span (Fig. S3), age class had a strong main effect (*P*<0.001 in both cases), confirming substantial baseline size differences between calves and adults (Fig S4). The interaction between flukeprint width and age class was not significant for either body length (*F*_1,88_=2.36, *P*=0.13) or fluke span (*F*_1,88_=0.76, *P*=0.39), indicating no statistical evidence that slopes differed between age classes. Nevertheless, these models substantially improved overall fit (body length adjusted *R*^2^=0.88; fluke span adjusted *R*^2^=0.77) by accounting for categorical size differences.

When regressions were performed separately within each age class, contrasting patterns emerged. Among adults (*n*=80), mean flukeprint width was positively associated with both body length (slope=1.46±0.28, *t*_78_=5.22, *P*<0.001) and fluke span (slope=0.41±0.12, *t*_78_=3.41, *P*=0.001). However, the proportion of variance explained within adults was modest (body length *R*^2^=0.25; fluke span *R*^2^=0.12), indicating that flukeprint width contains limited information about continuous variation in adult body size. In contrast, no relationship was detected within calves (*n*=12). Flukeprint width did not predict body length (slope=0.08±0.36, *t*_10_=0.22, *P*=0.83; *R*^2^=0.005) or fluke span (slope=0.08±0.11, *t*_10_=0.67, *P*=0.52; *R*^2^=0.04), with calf body size remaining effectively constant across the observed range of flukeprint widths (Fig. 3A). Ten-fold cross-validation of adult-only models confirmed limited predictive power (body length: RMSE=0.92 m, *R*^2^=0.38; fluke span: RMSE=0.38 m, *R*^2^=0.29), reinforcing that flukeprint width provides only coarse size information within adults.

Despite weak within-class scaling, flukeprint width proved to be a highly robust discriminator of age class. Logistic regression using mean flukeprint width alone resulted in a clear separation between calves and adults, reflected by an essentially zero residual deviance. ROC analysis yielded an optimal threshold of 2.49 m, with both sensitivity and specificity equal to 1.0, indicating perfect classification of calves and adults based on flukeprint width alone (Fig. 3B).

Flukeprint width contained a strong age-class signal, with calves and adults forming two distinct clusters relative to body length and fluke span. While pooled analyses suggested scaling between flukeprint width and morphology, this relationship was primarily driven by categorical differences between calves and adults rather than continuous variation across individuals. Within age classes, flukeprint width retained a weak but detectable association with adult body length and fluke span, whereas calf flukeprints varied more widely despite similar morphology. Rather than reflecting a measurement limitation, this contrast may reflect age-related differences in fluke compliance and propulsion mechanics, whereby ontogenetic changes in fluke stiffness, bending dynamics and stroke kinematics influence how fluke morphology translates into water displacement and surface wake. In calves, more flexible or less constrained flukes (Faria et al., 2013) could allow greater variation in lateral displacement during oscillatory motion, reducing the correspondence between morphology and flukeprint width. In adults, relatively stiffer flukes may produce more consistent lateral displacements, allowing flukeprint width to retain coarse information about body size. Environmental factors such as turbulence, currents and surface winds may further influence print formation, potentially adding variance to size-related inferences. Nonetheless, the clear age-class separation revealed by thermal flukeprints highlights their potential for non-invasive assessment of life-history stage in free-swimming whales. With larger datasets and concurrent measurements of fluke span and mechanical properties, flukeprint-based metrics could provide a novel opportunity to explore ontogenetic changes in propulsive efficiency and locomotor mechanics and their ecological consequences.

### Swimming speed

**Fig. 4.**
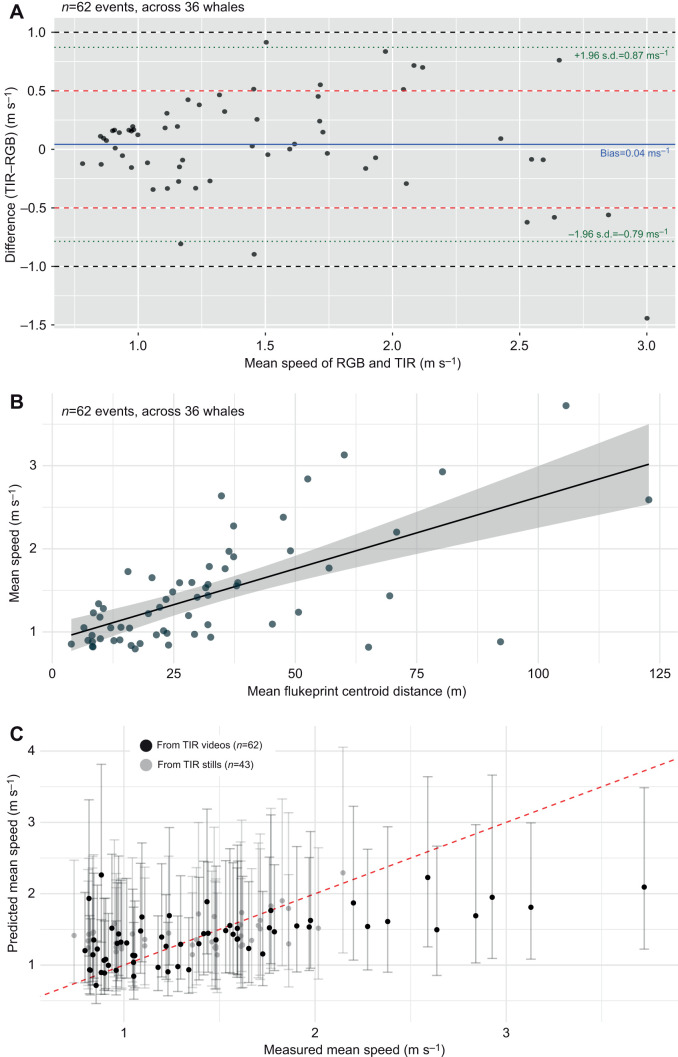
**Speed analysis of humpback whales from UAV imagery.** (A) Bland–Altman plot showing the agreement between RGB- and TIR-derived swimming speed. Each point represents the difference between RGB and TIR speed for a single measurement versus the mean speed. The solid blue line indicates the bias (mean difference). The dotted green lines indicate the limits of agreement (±1.96 s.d.) and the dashed lines indicate ±0.5 m s^−1^ (red) and ±1 m s^−1^ (black), as reference bounds. (B) Relationship between spacing of consecutive flukeprint centroids and swimming speed (TIR only, *n*=62 events across 36 whales). The shaded region represents the 95% confidence interval. (C) Comparison of predicted and observed whale speed. Black dots represent mean speed predicted from video-based tracking using leave-one-out cross-validation (LOOCV) with corresponding 95% confidence intervals (*n*=62 events across 36 whales). Grey dots represent mean speed predicted from flukeprint spacing in TIR-still images with 95% prediction intervals shown as vertical bars (*n*=43 events across 20 whales). The red dashed line indicates the 1:1 relationship, representing perfect agreement between predicted and observed speed.

Swimming speed was estimated for individual whales and surfacing events using frame-to-frame tracking of whale position in RGB imagery and centroid spacing of thermal flukeprints in TIR imagery. A total of 62 paired surfacing events across 36 whales were retained for comparison. The mean difference between RGB- and TIR-derived swimming speed was small (bias=0.04 m s^−1^; 95% confidence interval, CI: −0.15 to 0.07 m s^−1^) and there was no evidence of a systematic difference between estimates (paired *t*-test: *t*=−0.77, d.f.=59, *P*=0.44). Overall error was modest, with a RMSE of 0.42 m s^−1^ and a MAE of 0.31 m s^−1^. Across surfacing events, when scaled relative to observed swimming speeds, nRMSE was 0.28 and MAPE was 20.8%, with a median MAPE of 16.2%, indicating that typical relative discrepancies between RGB- and TIR-derived speed were of the order of 16–21%. Reliability between RGB- and TIR-derived estimates was moderate to high [ICC(A,1)=0.77; 95% CI: 0.65–0.86]. Bland–Altman analysis showed differences centred near zero, with 76.7% of paired estimates within ±0.5 m s^−1^ and 98.3% within ±1.0 m s^−1^ (Fig. 4A). Regression of paired differences against mean speed revealed no significant proportional bias (slope=−0.11, *P*=0.26), indicating that disagreement between methods did not systematically increase with swimming speed.

Analysis of TIR data revealed a positive association between mean spacing of consecutive thermal flukeprints and mean swimming speed (Fig. 4B). Correlation analyses indicated a moderate relationship (Pearson *r*=0.66; Spearman *P*=0.63). A log–log linear mixed-effects model confirmed that flukeprint spacing was a significant predictor of swimming speed (β=0.33±0.05 estimate±s.e., *t*=6.08, *P*<0.001), with whale identity included as a random intercept. The model explained 39.7% of the variance in swimming speed through fixed effects alone (marginal *R*^2^=0.40), increasing to 52.8% when including between-individual variation (conditional *R*^2^=0.53). Random intercept variance was small relative to residual variance, indicating limited inter-individual differences in the spacing–speed relationship. LOOCV showed moderate predictive performance (RMSE=0.52 m s^−1^; MAPE=26.0%), with 93.5% of observed speeds falling within the model's 95% prediction intervals. Comparison of predicted and observed speed indicated consistent under- and over-prediction across the observed speed range rather than random scatter (Fig. 4C).

Predicted swimming speeds derived solely from thermal flukeprint spacing in still images were positively associated with mean swimming speeds independently estimated from RGB imagery (*n*=43 surfacing events across 20 individuals). Model performance metrics indicated moderate predictive skill, with a RMSE of 0.34 m s^−1^, a MAPE of 21.3% and an *R*^2^ of 0.23. For those events, mean swimming speed estimated from RGB video sequences was 1.36 m s^−1^, while the corresponding mean speed predicted from thermal flukeprint spacing using still images was slightly higher at 1.43 m s^−1^. Consistent with this difference, prediction errors showed a small systematic bias, with a mean error of −0.08 m s^−1^, indicating a slight tendency to overestimate swimming speed at this range. All 43 surfacing events had observed mean swimming speeds falling within the corresponding 95% prediction intervals (Fig. 4C).

Thermal flukeprint spacing was positively associated with swimming speed, demonstrating that surface thermal signatures can serve as practical, non-invasive indicators of horizontal locomotion. TIR-derived speed aligned with previously reported average humpback swimming speed in breeding grounds (0.8–1.1 m s^−1^; Kela et al., 2024) and comparisons with RGB-derived speed showed close correspondence at the level of individual surfacing events, with low bias and moderate relative error. Discrepancies were proportional to speed rather than systematic overestimation or underestimation, reflecting heteroscedasticity at higher velocities. The relationship between flukeprint spacing and speed was strongest at lower velocities, where tailbeat frequency and amplitude produced consistent displacement, and became more variable at higher speeds, probably due to behavioural flexibility and combinations of stroke amplitude, frequency and gliding. As a result, thermal flukeprint spacing provides a more constrained proxy for swimming speed at low velocities and a coarser, though still informative, indicator at higher speeds, capturing the net effect of locomotor mechanics in a non-invasive way. Cross-validation of the mixed-effects model reflected these limitations: predictive performance was generally good, with 93.5% of observed speeds falling within the 95% prediction intervals, but precision declined at the extremes, with slight overestimation at low speeds and underestimation at high speeds.

Wider spacing between consecutive thermal flukeprints being positively associated with higher swimming speeds was consistent with fundamental principles of cetacean locomotion, whereby horizontal velocity reflects interacting effects of tailbeat amplitude and frequency (Ayancik et al., 2020; Fish, 1998; Han et al., 2020). Tag-based studies have demonstrated that whales can achieve faster swimming through different combinations of these parameters, including increases in stroke amplitude, tailbeat frequency or both (Gough et al., 2019). In the present study, thermal flukeprint spacing did not resolve individual tailbeats or stroke kinematics; instead, it integrated the cumulative effect of propulsion between surfacing events into a single, surface-visible measure of net horizontal displacement. In future work, incorporating finer-scale thermal signatures generated by sub-surface tailbeats between surfacings may allow partial resolution of propulsion frequency and improve discrimination among locomotor strategies. Taken together, these results suggest that, with the approach used in this study, thermal flukeprint spacing is best interpreted as an integrative, surface-based metric of swimming performance rather than a precise estimator of instantaneous speed. Within this context, the method provides a practical means of quantifying relative swimming speed in situations where animal-borne sensors are impractical, while also highlighting opportunities for future work to improve resolution at higher velocities.

### Orientation

**Fig. 5.**
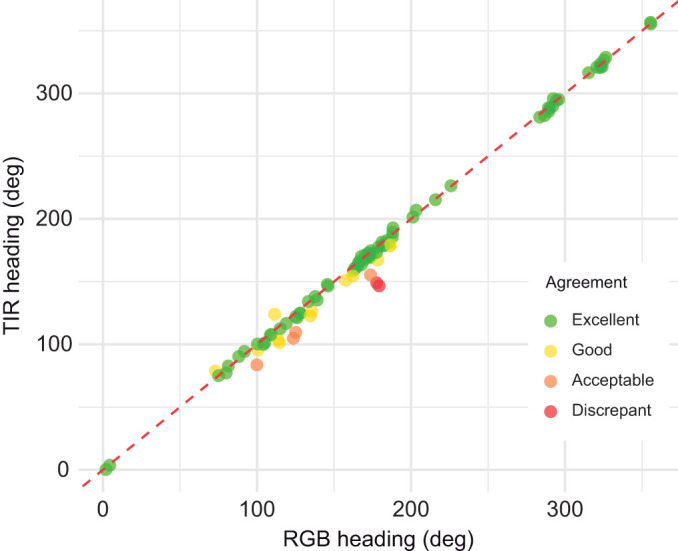
**Orientation analysis of humpback whales from UAV imagery.** Agreement between heading measurements from TIR and RGB imagery, classified by agreement strength: Excellent (≤ 5 deg), Good (≤ 15 deg), Acceptable (≤ 20 deg) and Discrepant (>20 deg). The red dashed line represents perfect agreement (1:1 relationship).

A total of 91 paired heading estimates were obtained across 23 individual whales. When pooling across all observations, the circular mean orientations were 158.2 deg for RGB and 152.2 deg for TIR, both with low within-sensor circular standard deviations (1.30 deg and 1.32 deg, respectively). These pooled means should be understood as statistical summaries for comparing modalities, rather than as predominant travel directions of the whales. The circular correlation between TIR- and RGB-derived headings was very high (*r*=0.98, *P*<0.0001). Paired difference analysis indicated a mean offset of −3.1 deg (TIR−RGB) with a global standard deviation of 6.4 deg, and the differences were significantly clustered around this mean (Rayleigh test: *Z*=0.99, *P*<0.001), indicating that TIR and RGB headings were consistently aligned rather than randomly varying relative to each other (Fig. S5). Agreement classification further confirmed the close correspondence between methods: 80.2% of TIR estimates fell within 5 deg of the RGB reference (Excellent), 13.2% within 15 deg (Good) and 4.4% within 20 deg (Acceptable), with only 2.2% showing slightly larger discrepancies just above 20 deg (i.e. <24 deg) (Fig. 5).

Orientation estimates derived from TIR footage showed close correspondence with RGB-based reference values, with differences typically less than 5 deg, demonstrating that thermal flukeprints preserve not only displacement and scale but also fine-scale directional information. This ability to track heading extends the utility of TIR imaging for reconstructing movement trajectories, particularly when animals are only intermittently visible at the surface. When combined with speed estimates, these measurements could facilitate two-dimensional path reconstruction from flukeprints alone, helping to bridge the gap between fine-scale kinematic studies based on animal-borne sensors (Wright et al., 2017) and broader-scale movement ecology inferred from satellite telemetry (Cubaynes et al., 2019). While our analyses focus on orientation and short-term movement inferred from consecutive flukeprints, the approach has the potential to extend to larger spatial and temporal scales.

### Limitations and future directions

By validating TIR-derived metrics against RGB reference data and established principles of whale locomotion, we show that surface-visible thermal traces carry biological signals that can be leveraged to study fine-scale behaviour under conditions where conventional methods are limited (Stewart et al., 2024; Thomas and Thorne, 2001). While thermal flukeprints provide a novel and non-invasive window into whale morphology and movement, several limitations should be considered when interpreting the results of this study.

First, thermal flukeprints are transient features whose geometry evolves as a result of mixing, diffusion and ambient water motion. Although prints were measured at their earliest clearly defined boundary following fluke emergence, typically within seconds of the tailbeat, some temporal variability in flukeprint size and shape is unavoidable and probably contributes to measurement noise, particularly for fine-scale morphometric inference. Future studies combining thermal imagery with controlled observations of wake decay or concurrent hydrodynamic measurements could help quantify flukeprint persistence and improve temporal standardization. Diffusion and overlap of prints, especially in groups of interacting whales, can further obscure individual trajectories. Environmental drivers of print persistence and detectability have not yet been systematically evaluated and incorporating factors such as current velocity, sea state and wind conditions would probably refine predictions of speed and orientation. More generally, systematic characterization of detection limits and persistence, together with model refinement using environmental covariates and exploration of finer-scale thermal signals within individual prints, may yield additional insights into tailbeat dynamics, stroke energetics and intra-sequence variation in locomotor effort, thereby expanding the utility of TIR-based methods for addressing broader ecological and behavioural questions, including energy expenditure, predator–prey interactions and the role of locomotion in social and reproductive contexts (Blawas et al., 2025; Spina et al., 2024; Wright et al., 2017).

Second, hydrodynamic complexity increases at higher swimming speeds. While thermal flukeprint spacing showed a clear relationship with horizontal swimming speed at low to moderate speeds, this relationship weakened at higher speeds, where additional factors such as thrust orientation, acceleration, depth-dependent drag and non-steady swimming behaviour become increasingly important. Because the present approach does not directly measure thrust magnitude, flow velocity fields or the orientation of propulsive forces, speed estimates derived from flukeprint spacing should be interpreted as coarse indicators of swimming state rather than precise locomotor metrics. Integrating thermal imaging with particle image velocimetry, stereo imagery or biologging sensors represents a promising direction for resolving these dynamics. In addition, expanding datasets to include individuals spanning a wider range of body sizes may help clarify how ontogenetic differences in fluke compliance and thrust generation influence spacing–speed relationships, an effect that could not be evaluated in the present study but represents an important avenue for future work.

Third, positional uncertainty can contribute to error in swimming speed estimates derived from RGB imagery. Whale position was tracked using a consistent anatomical landmark (the tip of the rostrum) across consecutive frames, minimizing ambiguity associated with partial body visibility. Residual error arises from image georeferencing accuracy and manual landmark identification, but this error is expected to be small relative to frame-to-frame displacements during linear swimming and was further reduced by averaging multiple consecutive measurements within surfacing events. As a result, positional uncertainty is unlikely to materially affect comparisons between RGB- and TIR-derived speed estimates, though it may limit fine-scale kinematic inference.

Finally, several promising extensions of this approach remain to be explored. Differences in fluke compliance, thrust production and wake structure between calves and adults may have important implications for locomotor efficiency, maternal investment and migratory behaviour, but addressing these points will require larger datasets and direct measurement of fluke span, stiffness and wake energetics. Similarly, thermal imaging holds strong potential for nocturnal and low-visibility monitoring, as it does not rely on ambient light and can reveal thermal contrasts that are invisible in RGB imagery. Systematic validation of performance under these conditions represents an important and promising direction for future work and will further expand the scope of applications of this approach.

Taken together, these limitations highlight both the current scope of thermal flukeprint analysis and its considerable potential. As thermal imaging technology and analytical methods continue to advance, combining flukeprint-based inference with complementary biomechanical and hydrodynamic data will enable increasingly detailed insights into the movement ecology of large marine vertebrates.

### Conclusion

This study contributes to the development of scalable, ethical tools for marine mammal research and conservation, providing non-invasive access to fundamental biological metrics such as age class discrimination, speed estimates and orientation. By capturing surface-visible thermal traces that encode locomotor and ecological information, TIR imagery complements established approaches such as tagging and satellite telemetry, while overcoming several of their practical limitations. While further work is needed to validate performance under conditions such as night-time or turbid waters, this technique has the potential to support population-level and long-term monitoring in such challenging environments, as well as in populations where tagging is unfeasible. As marine environments face increasing pressure from climate change, vessel traffic and offshore development, approaches such as thermal print analysis support long-term ecological data collection while minimizing disturbance to wildlife. Overall, this method represents a significant step toward more comprehensive, less intrusive studies of whale behaviour, energetics and ecology, with clear applications in conservation, management and fundamental biology.

## Supplementary Material

10.1242/jexbio.251816_sup1 Supplementary information
