# Supplementary material for: In the tracks of a whale: inferring size class, orientation and swimming speed from thermal flukeprints
Source: J Exp Biol. 2026 Apr 17;229(8):jeb251816. doi: 10.1242/jeb.251816 (PMC13120677; doi:10.1242/jeb.251816)
Supplement: Supplementary information [file jexbio-229-251816-s1.pdf]

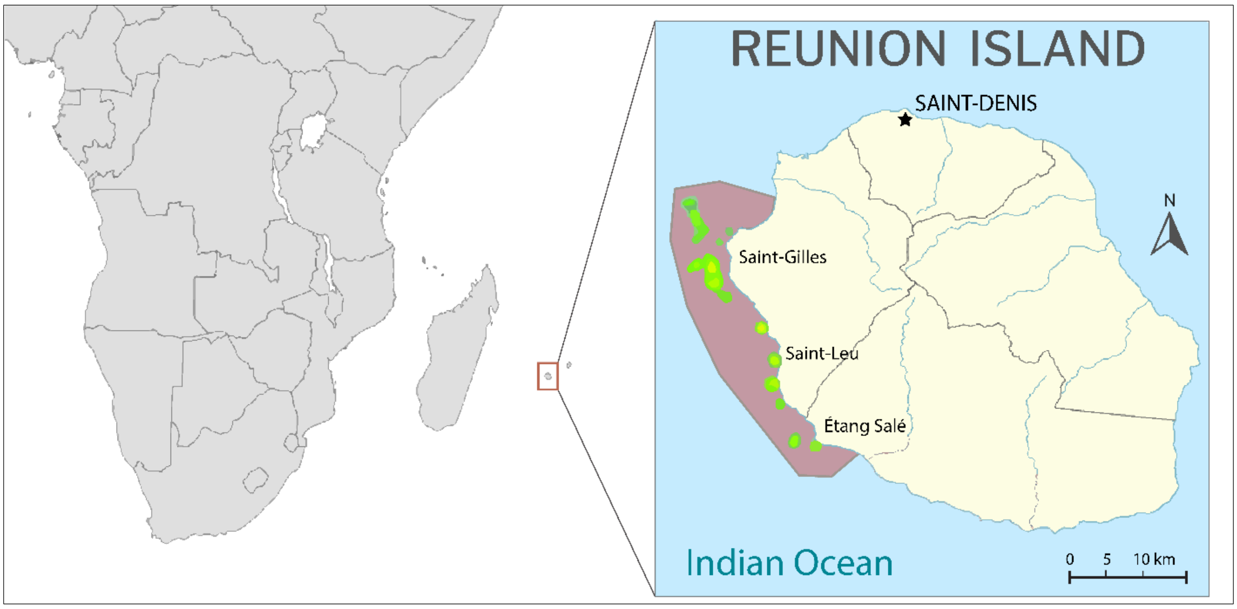

Fig. S1. Geographic location of Reunion Island in the southwest Indian Ocean (left). The inset map shows the island with the study area highlighted in red along the west coast. Green heat map points indicate the locations where drone surveys were conducted to collect flukeprint data and behavioural observations of humpback whales using RGB and TIR imagery.

Journal of Experimental Biology • Supplementary information

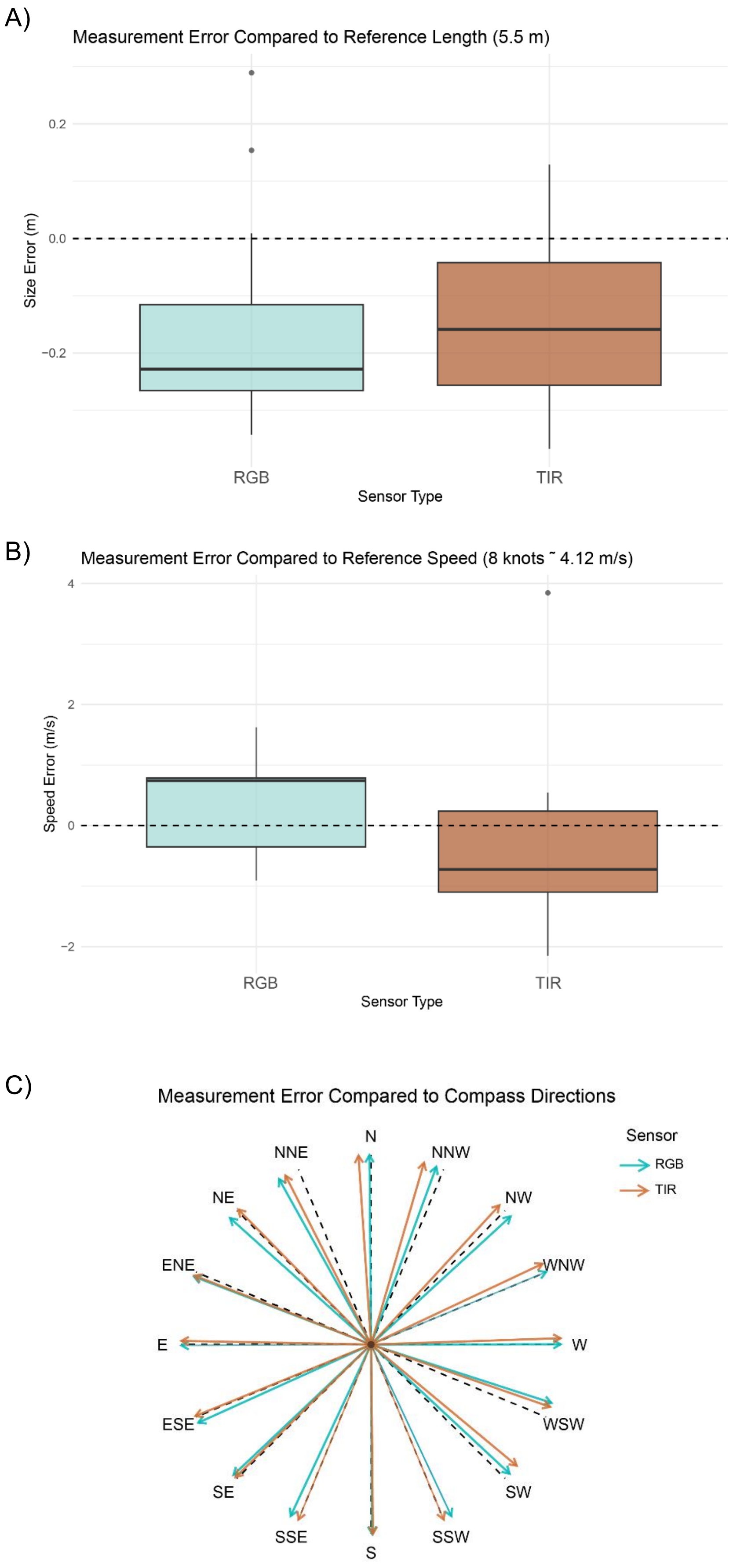

Fig. S2. Absolute measurement of error between reference values and DVM-measured metrics for (A) size, (B) speed, and (C) orientation.

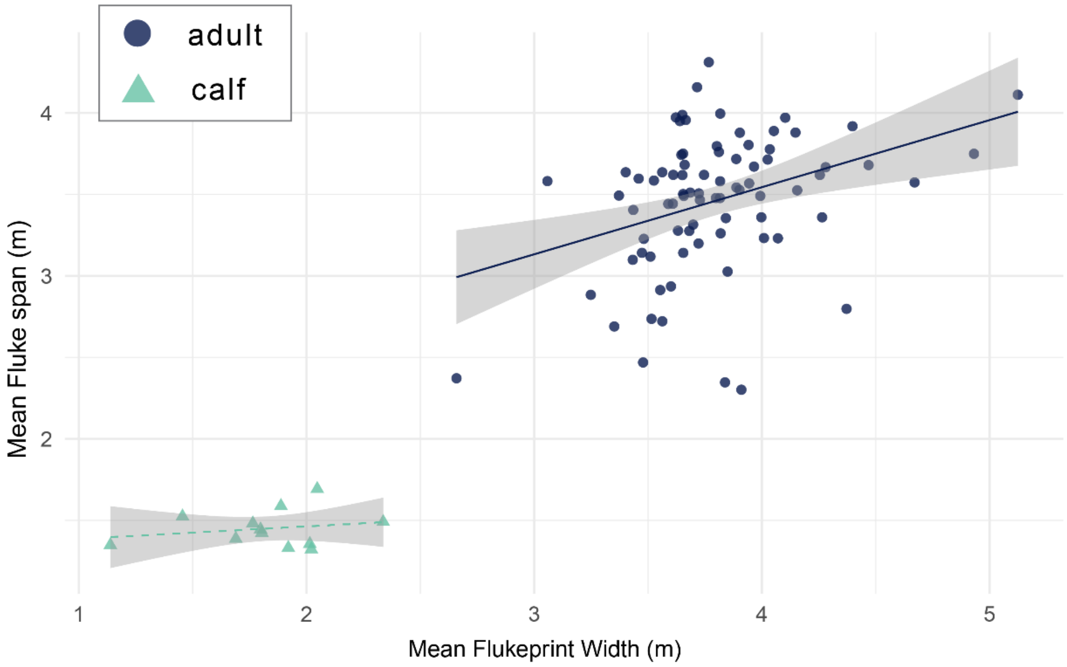

Fig. S3. Fluke span to flukeprint width for all whales included in the size analysis (n adults = 80, n calves = 12).

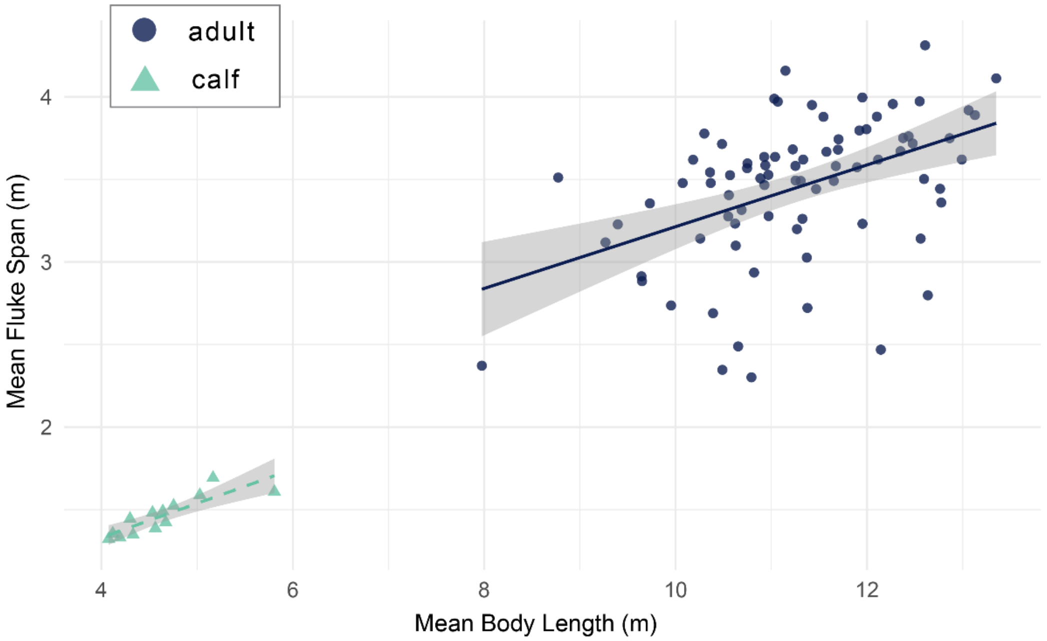

Fig. S4. Fluke span to body length for all whales included in the size analysis (n adults = 80, n calves = 12).

Journal of Experimental Biology • Supplementary information

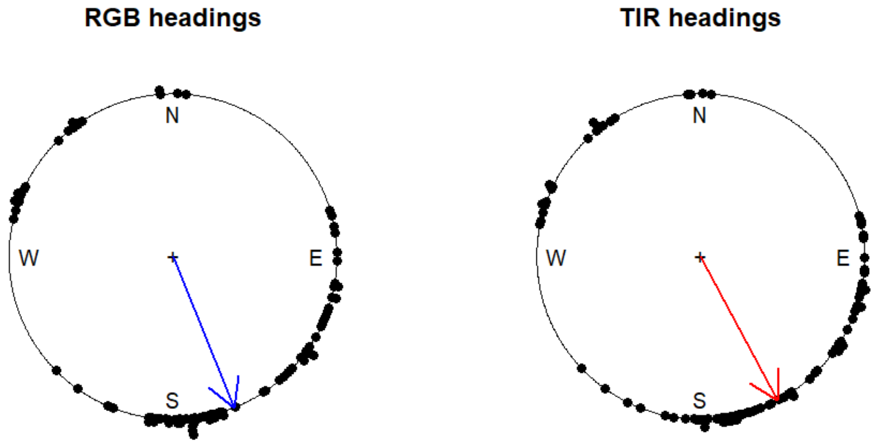

Fig. S5. Individual movement directions and overall pooled averages derived from the animals (RGB; pooled average shown in blue), and from flukeprints (TIR; pooled average shown in red).

Journal of Experimental Biology • Supplementary information

Example 1

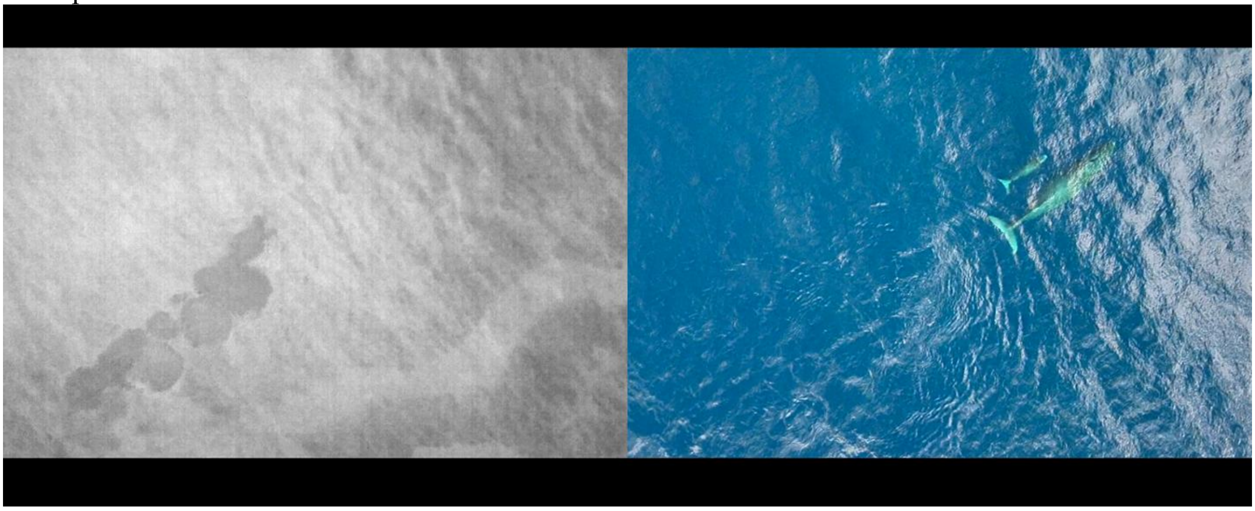

Example 2

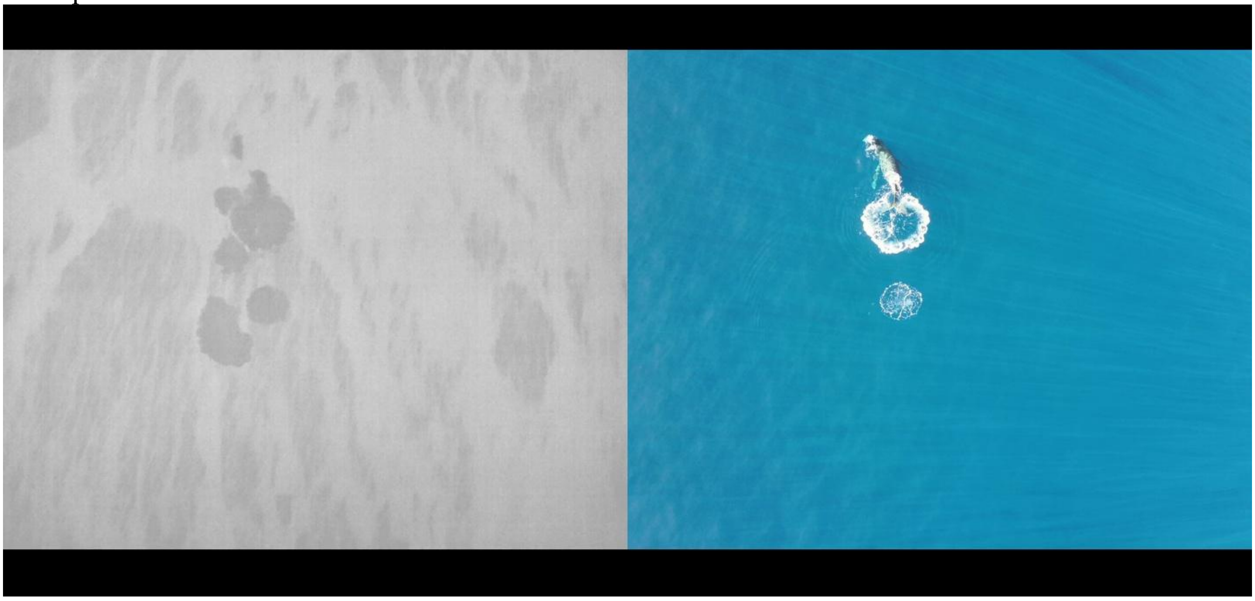

Fig. S6. Examples of synchronized TIR-RGB frames showing size comparison between mother–calf pairs**.**

Journal of Experimental Biology • Supplementary information

Example 1

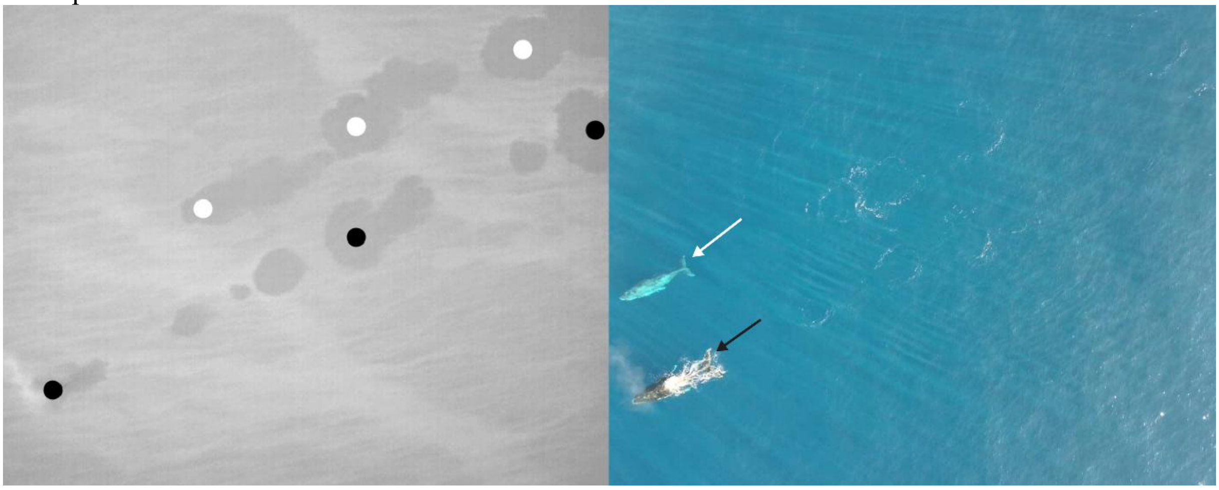

Example 2

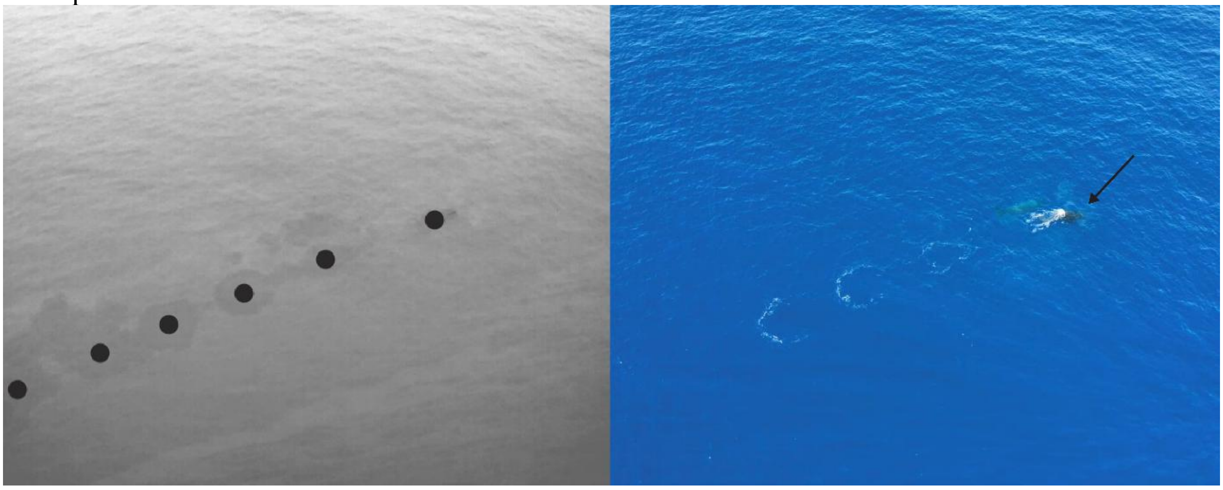

Example 3

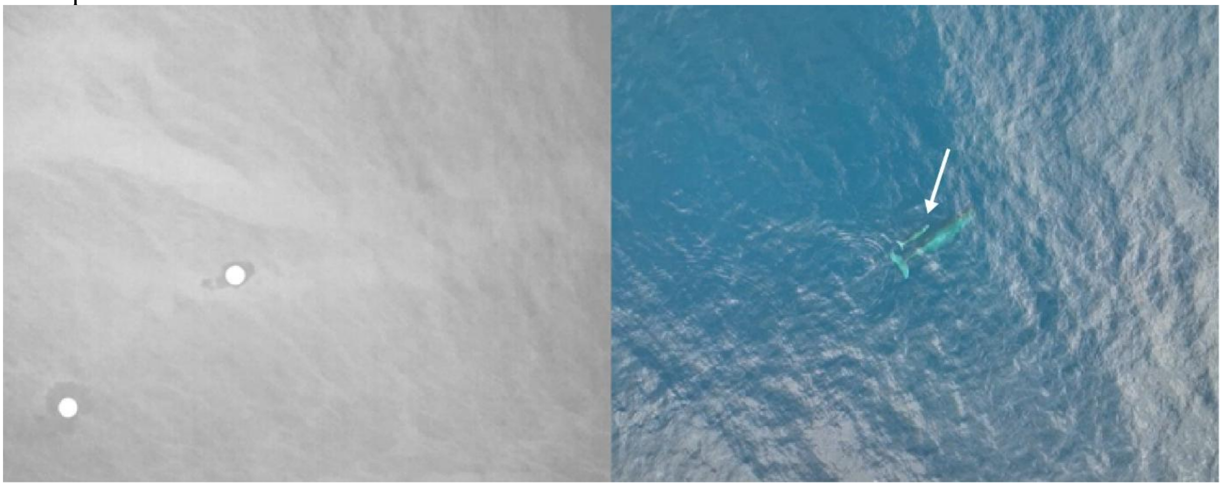

Fig. S7. Still frames illustrating flukeprint spacing used to estimate swimming speed. Dots in the TIR frames (left) mark flukeprint centroids, and arrows in the RGB frames (right) link each sequence to its corresponding whale; black and white shading distinguishes individual whales.

Journal of Experimental Biology • Supplementary information

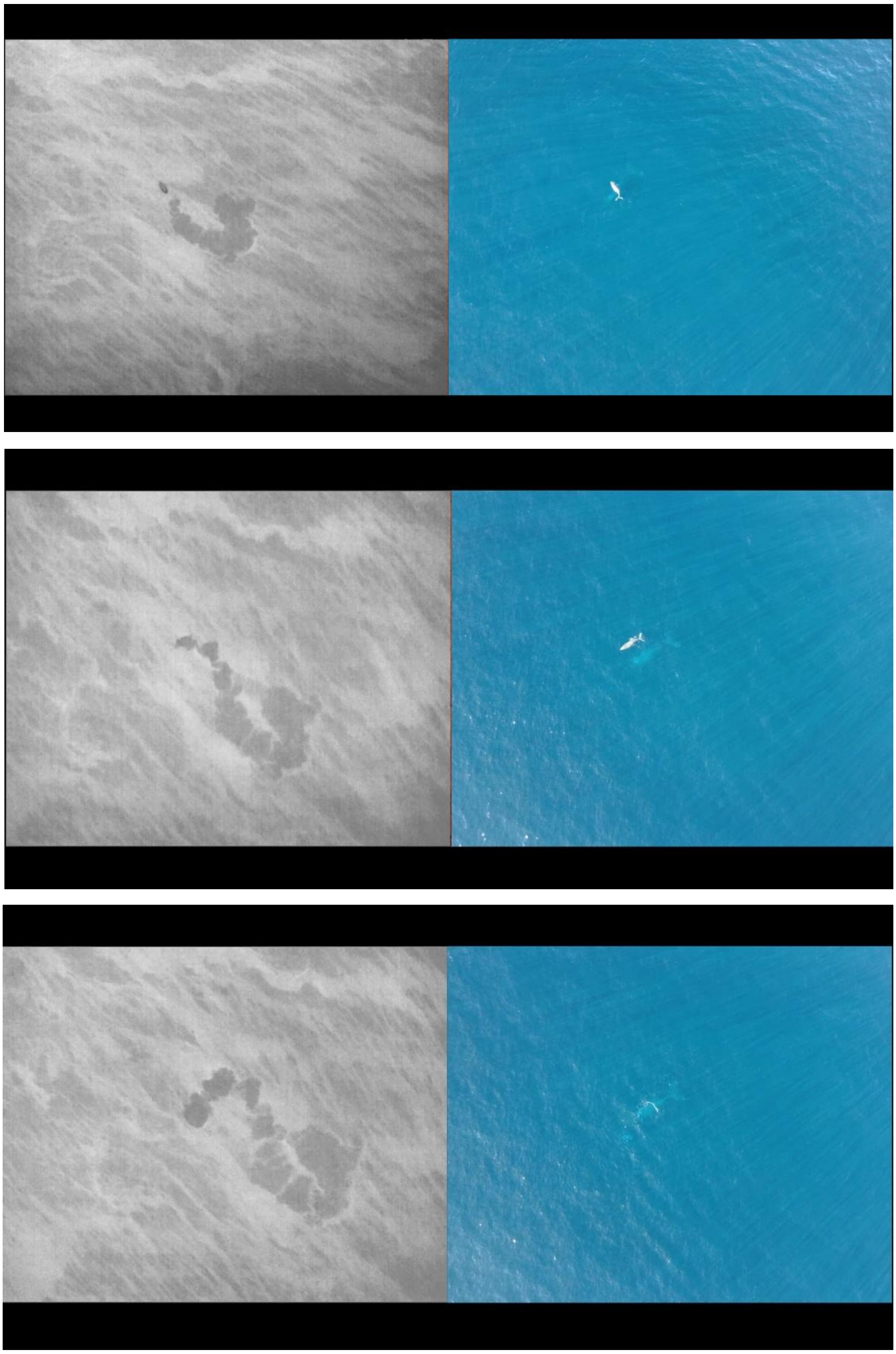

Fig. S8. Three consecutive video frames showing a whale's changing headings inferred from flukeprints. The thermal tracks reveal successive directional changes and the short-term history of movement.

Journal of Experimental Biology • Supplementary information

Table S1. Accuracy assessment of DVM-derived measurements for size, speed, and orientation. Reference values were obtained from a physical object of known dimensions (size) and from onboard sensors (speed and orientation). For each sensor, measurements are reported in the same units as their respective reference values. Absolute error is given in reference-unit values, and relative error (%) is shown in parentheses. Orientation errors are expressed as circular deviations relative to reference compass bearings.

		TIR		RGB	
**Parameters**	**Reference value**	**Measurement**	**Error**	**Measurement**	**Error**
**size** (m)	5.5	5.35 ± 0.15	−0.15, *(-2.8%)*	5.34 ± 0.15	−0.16, *(-2.8%)*
**speed** (m s^{-1})	4.1	4.05 ± 0.07	−0.07, *(−1.7%)*	4.49 ± 0.38	+0.38, *(+9.2%)*
**orientation** (°)	Overall (0-360)	NA	2.1, *(0.6%)*	NA	2.0, *(0.5%)*
	N (0)	3.8	3.8	0.5	0.5
	NNE (22.5)	26.8	4.3	29.0	6.5
	NE (45)	44.5	0.5	48.1	3.1
	ENE (67.5)	68.6	1.1	69.0	1.5
	E (90)	88.9	1.1	90.2	0.2
	ESE (112.5)	112.1	0.3	114.4	1.9
	SE (135)	134.4	0.6	133.5	1.5
	SSE (157.5)	157.5	0.0	154.8	2.6
	S (180)	180.6	0.6	180.3	0.3
	SSW (202.5)	202.5	0.0	205.1	2.6
	SW (225)	230.2	5.2	227.0	1.9
	WSW (247.5)	250.8	3.3	252.1	4.6
	W (270)	271.9	1.9	270.1	0.1
	WNW (292.5)	295.2	2.7	292.5	0.0
	NW (315)	317.4	2.4	312.6	2.4
	NNW (337.5)	343.8	6.3	339.8	2.3

Journal of Experimental Biology • Supplementary information

Table S2. Output from linear and linear mixed models predicting swimming speed. Estimates are given on the log–log scale, with standard errors (SE), t-values, and REML/R^2 for model fit. Back-transformed interpretations are provided for biological meaning.

Model	Predictor	Estimate	SE	t-value	REML/R^2	Note
**LMM (with ID)**	(Intercept)	-0.775	0.183	-4.24	REML = 35.2	Accounts for individual variation
	spacing	0.333	0.055	6.08	-	significant
**LMM (ID + body_length)**	(Intercept)	0.789	0.242	-3.27	REML = 34.4	Includes body length as fixed effect
	spacing	0.353	0.057	6.15	-	Significant; spacing effect remains
	body_length	-0.025	0.121	-0.205	-	Not significant: no effect of body length

Back-transformed interpretation:

LMM (ID): speed ~ spacing + (1 | whaleID)
exp(-0.775)=0.46 m/s → expected speed at spacing = 1 m
A 1% increase in spacing → 0.33% increase in speed

Journal of Experimental Biology • Supplementary information
